# Sport Expertise and Twitch-Derived Agonist–Antagonist Contractile Ratio in Karate Athletes

**DOI:** 10.3390/jfmk11020152

**Published:** 2026-04-14

**Authors:** Velimir Jeknic, Milivoj Dopsaj, Nenad Koropanovski

**Affiliations:** 1 Faculty of Sport and Physical Education, University of Belgrade, 11030 Belgrade, Serbia; milivoj.dopsaj@gmail.com; 2 Department of Criminalistics, University of Criminal Investigation and Police Studies, 11080 Belgrade, Serbia; nenad.koropanovski@kpu.edu.rs

**Keywords:** karate, tensiomyography index, agonist-antagonist coupling, muscle mechanics, neuromuscular adaptation

## Abstract

**Objectives**: Agonist–antagonist coordination is traditionally defined as simultaneous neural activation assessed by electromyography (EMG). The present study adopts a mechanical perspective, examining twitch-derived contractile ratio indexes between antagonistic muscle groups using tensiomyography (TMG). The aim was to determine whether sport expertise differentiates mechanical agonist–antagonist coordination in karate athletes. **Methods**: Fifty male participants were divided into four groups: elite karate athletes (EK; *n* = 7), national team members (NK; *n* = 14), basically trained karate practitioners (BK; *n* = 16), and physically active non-athlete controls (CG; *n* = 13). Bilateral TMG assessment of rectus femoris, vastus lateralis, vastus medialis, biceps femoris, and semitendinosus was performed. Contraction time (Tc), total contraction time (TcT), and rate of muscle tension development (RMTD) were extracted. Twelve twitch-derived contractile ratio indexes (CRI) were calculated separately for dominant (D) and non-dominant (ND) limbs. **Results**: Significant between-group differences were observed in the temporal coordination of the non-dominant leg. EK demonstrated the lowest index for average contraction time (CRI_Tc_AVG_ND = 17.13%; ANOVA *p* = 0.005; EK vs. NK *p* = 0.003) and total contraction time (CRI_TcT_AVG_ND = 9.72%; ANOVA *p* = 0.003; EK vs. NK *p* = 0.002). In contrast, velocity-related coordination in the dominant leg was highest in EK (CRI_RMTD_cV_D = 63.66%; ANOVA *p* = 0.002), differing from NK (*p* = 0.003), BK (*p* = 0.002), and CG (*p* = 0.009). **Conclusions**: Elite karate athletes exhibit distinct twitch-derived mechanical coordination profiles characterized by highly efficient temporal interplay in the non-dominant (supportive) limb and elevated velocity-related contractile ratio in the dominant (executive) limb. These findings suggest that sport expertise is associated with task-specific mechanical modulation between antagonistic muscle groups detectable through involuntary contractile responses.

## 1. Introduction

Efficient coordination between agonist and antagonist muscles is fundamental to dynamic movement control, joint stability, and performance optimization in sport [1,2]. In classical neuromuscular physiology, coactivation refers to the simultaneous activation of agonist and antagonist muscles and is typically quantified using electromyography (EMG) [3,4,5]. This neural coactivation influences joint stiffness, movement precision, and mechanical efficiency [6,7].

However, neuromuscular coordination can also be explored from a mechanical perspective [8]. Instead of measuring neural activation directly, the present study evaluates twitch-evoked contractile responses using tensiomyography (TMG). In this context, the ratio-based mechanical relationship reflects the relative contractile behavior between antagonistic muscle groups (knee extensors and flexors), derived from twitch responses rather than voluntary activation. Unlike EMG-based coactivation, which quantifies simultaneous neural drive, these ratios represent the balance of intrinsic muscle mechanical properties such as contraction timing and contraction velocity. Therefore, the proposed indexes provide an indirect but functionally meaningful descriptor of mechanical intermuscular coordination, where the temporal interplay between opposing muscle groups is critical for performance and joint stability.

In striking-based combat sports such as karate, precise muscles agonist–antagonist interplay is critical [9]. During hand strikes, the lower body contributes primarily because ground reaction force is generated from the legs and transmitted to the upper body, enabling a powerful movement [10]. Kicking techniques require rapid knee extension combined with controlled deceleration [11], while the non-dominant leg must maintain dynamic stability during single-leg stance [12]. Elite performance, therefore, depends not only on contraction speed or force magnitude but also on optimized temporal and velocity-related coordination between opposing muscle groups [13].

Tensiomyography provides a non-invasive method for assessing contractile muscle properties via electrically evoked twitch responses, offering advantages such as selective muscle assessment and minimal influence of central fatigue [14]. Parameters such as contraction time (Tc), total contraction time (TcT), and rate of muscle tension development (RMTD) reflect temporal and velocity characteristics of muscle behavior independent of voluntary motor command [15]. While TMG has been widely used to describe muscle contractile properties across sports [16,17,18], its potential to quantify intermuscular mechanical coordination through agonist–antagonist ratios remains insufficiently explored. Also, previous research suggests that higher sport expertise is associated with refined neuromuscular strategies, including optimized balance control and improved modulation of antagonist activity during explosive tasks [19,20,21]. Nevertheless, it remains unclear whether involuntary, twitch-derived contractile behavior reflects expertise-dependent coordination patterns.

Accordingly, the aim of this study was to investigate whether sport expertise is associated with differences in twitch-derived mechanical coordination indexes of knee extensors and flexors, obtained using tensiomyography. Specifically, we (1) present basic TMG contractile parameters (Tc, TcT, and RMTD) across groups of differing skill level, and (2) compare mechanical agonist–antagonist contractile ratio indexes between elite, national-level, basically trained, and non-athlete participants. We hypothesized that elite athletes would demonstrate more efficient temporal coordination in the non-dominant (supportive) leg and distinct velocity-related coordination patterns in the dominant (executive) leg.

## 2. Materials and Methods

### 2.1. Participants

The sample comprised four male groups: elite karate athletes (EK; *n* = 7), national karate team athletes (NK; *n* = 14), basically trained karate practitioners (BK; *n* = 16), and a control group (CG; *n* = 13). All participants were free from neuromuscular, orthopedic, or metabolic disorders and reported no injuries within the previous six months that could affect testing performance. The inclusion criteria for the abovementioned groups were as follows: EK were national team members who won medals in World or European Championships in the last two years; NK were national team members who participated in World or European Championships in the last two years but have not won any medal; BK are students who had basic karate training in the duration of 6 months; and CG are healthy, physically active non-athletes matched for age and sex with other subgroups. For the EK, NK, and BK groups, the dominant leg (D) was identified as the limb primarily used for kicking and propulsive movement in fighting stance, while the non-dominant leg (ND) served a stabilizing and balance-maintaining role during technical execution [11]. For the control group (CG), lower-limb dominance was established using the Waterloo Footedness Questionnaire-Revised [22]. All participants were informed about the purpose and procedures of the study and signed a written consent form. Ethical approval was obtained from the Ethics Committee of the Faculty of Sport and Physical Education, University of Belgrade (Ref. No. 484-2), in accordance with the Declaration of Helsinki.

### 2.2. Experimental Procedures

Testing took place in the morning hours under controlled laboratory conditions (22–24 °C). Participants refrained from strenuous physical activity for 48 h before testing and from consuming caffeine, alcohol, or any stimulants on the day of measurement. Each session consisted of anthropometric assessment followed by evaluation of involuntary muscle contractile properties via tensiomyography (TMG). Anthropometric measures were obtained using a stadiometer (±0.1 cm) and a calibrated digital scale (±0.1 kg); body mass index (BMI) was calculated as BM/BH^2^. Descriptive data for body height (BH), body mass (BM), and BMI are presented as mean ± standard deviation (SD)—Table 1.

TMG recordings were obtained using a TMG-100 electrostimulator (TMG-BMC d.o.o., Ljubljana, Slovenia) with a GK-40 displacement sensor (Panoptik, Ljubljana, Slovenia), following standardized procedures [23]. The examined muscles included the rectus femoris (RF), vastus medialis (VM), vastus lateralis (VL), biceps femoris (BF), and semitendinosus (ST) on both limbs. Participants were positioned supine for testing the RF, VM, and VL, and prone for testing the BF and ST. The knee joint angle was fixed at 120° to ensure standardized muscle length across participants and measurement conditions. This position is commonly used in TMG assessments [15], as it provides a stable and reproducible mechanical configuration, minimizing variability in muscle slack and optimizing the reliability of contractile parameter extraction. The measurement site for each muscle was identified as the point of maximal belly displacement during a voluntary contraction, verified by palpation. Self-adhesive stimulation electrodes were placed longitudinally over the muscle belly, with the sensor positioned equidistantly between them. Each muscle received six single electrical impulses (1 ms duration; 10–110 mA, in 25 mA increments) with a 10 s inter-stimulus interval to prevent potentiation and fatigue effects [24]. The trial producing the maximal displacement (Dm) was used for analysis.

For each tested muscle, the following mechanical variables were extracted from the TMG displacement curve:

• Dm (mm)—maximum radial displacement of the muscle belly.
• Td (ms)—delay time from stimulus to 10% of Dm.
• Tc (ms)—contraction time from 10% to 90% of Dm.

Subsequently, the following TMG parameters were calculated:

• TcT (ms)—total contraction time, computed as Td + Tc.
• RMTD (mm/ms)—rate of muscle tension development, calculated as Dm/Tc [25]. This parameter reflects the speed at which the muscle reaches its maximal radial displacement during an involuntary twitch response, making it the closest TMG-derived indicator of contraction velocity.

### 2.3. Calculation of Contractile Ratio Indexes

Mechanical coordination was calculated as a ratio-based relationship between twitch-derived contractile parameters of the knee extensor and flexor muscles. Contractile ratio indexes (CRI) reflect relative mechanical behavior between antagonistic muscle groups and do not represent simultaneous neural activation as assessed by electromyography. Lower values indicate greater similarity in contractile timing between opposing muscles, whereas higher values reflect greater mechanical disparity. To evaluate neuromuscular mechanical coordination between agonist and antagonist muscle groups, 12 contractile ratio indexes were computed for every group based on TMG variables’ average and coefficient of variation perspective of the Tc, TcT, and RMTD [26]:

(1) $$CRI\_Tc\_AVG=\left(\frac{\left(BF_{Tc}+\;ST_{Tc}\right)\;÷\;2}{\left(RF_{Tc}\;+\;VL_{Tc}\;+\;VM_{Tc}\right)\;÷3}\times 100\right)-100$$

(2) $$CRI\_Tc\_cV=\left(\frac{SD\;\left(BF_{Tc},\;ST_{Tc}\right)\;÷\;AVG\;\left(BF_{Tc},\;\;ST_{Tc}\right)\times 100}{SD\;\left(RF_{Tc},\;\;\;VM_{Tc},\;\;\;VL_{Tc}\right)\;÷\;AVG\;\left(RF_{Tc},\;\;\;VM_{Tc},\;\;\;VL_{Tc}\right)\times 100}\times 100\right)-100$$

(3) $$CRI\_TcT\_AVG=\left(\frac{\left(BF_{TcT}+ST_{TcT}\right)\;÷\;2}{\left(RF_{\mathrm{TcT}}+VL_{\mathrm{TcT}}+VM_{\mathrm{TcT}}\right)\;÷3}\times 100\right)-100$$

(4) $$CRI\_TcT\_cV=\left(\frac{SD\;\left(BF_{TcT},\;\;\;ST_{TcT}\right)\;÷\;AVG\;\left(BF_{TcT},\;\;\;ST_{TcT}\right)\times 100}{SD\;\left(RF_{TcT},\;\;VM_{TcT},\;\;VL_{TcT}\right)\;÷\;AVG\;\left(RF_{TcT},\;\;\;VM_{TcT},\;\;\;VL_{TcT}\right)\times 100}\times 100\right)-100$$

(5) $$CRI\_RMTD\_AVG=\left(\frac{\left(BF_{RMTD}+ST_{RMTD}\right)\;÷\;2}{\left(RF_{RMTD}+VL_{RMTD}+VM_{RMTD}\right)\;÷3}\times 100\right)-100$$

(6) $$CRI\_RMTD\_cV=\left(\frac{SD\;\left(BF_{RMTD},\;\;\;ST_{RMTD}\right)\;÷\;AVG\;\left(BF_{RMTD},\;\;\;ST_{RMTD}\right)\times 100}{SD\;\left(RF_{RMTD},\;\;\;VM_{RMTD},\;\;\;VL_{RMTD}\right)\;÷\;AVG\;\left(RF_{RMTD},\;\;\;VM_{RMTD},\;\;\;VL_{RMTD}\right)\times 100}\times 100\right)-100$$

These indexes quantify temporal (Tc and TcT) and velocity-based (RMTD) ratios between extensors (RF, VL, and VM) and flexors (BF and ST) for both dominant and non-dominant limbs. Lower index values indicate more efficient agonist–antagonist coordination, whereas higher values suggest greater overlap. In this way, the area of neuromuscular contractile ratio of the knee joint of the dominant and non-dominant legs was described with 12 variables (CRI_Tc_AVG_D, CRI_Tc_cV_D, CRI_Tc_AVG_ND, CRI_Tc_cV_ND, CRI_TcT_AVG_D, CRI_TcT_cV_D, CRI_TcT_AVG_ND, CRI_TcT_cV_ND, CRI_RMTD_AVG_D, CRI_RMTD_cV_D, CRI_RMTD_AVG_ND, and CRI_RMTD_cV_ND), in four groups of subjects different in relation to the level of adoption of the karate skill—EK, NK, BK, and CG.

### 2.4. Statistical Analysis

The Shapiro–Wilk test was performed, which showed that the distribution of all variables included followed a normal distribution. One-way ANOVA determined between-group differences for EK, NK, BK, and CG, and the T-test was used to determine the differences between pairs of variables. The effect of the difference between the groups was determined by application of the Bonferroni correction, where the value of Eta^2^ was calculated, as well as the power of the analysis. All analyses were performed using IBM SPSS Statistics v. 20.0 (IBM Corp., Chicago, IL, USA). Statistical significance was set at *p* < 0.05.

## 3. Results

From a practical perspective, the most notable findings include: (1) significantly lower temporal contractile ratio indexes (Tc and TcT) in the non-dominant leg of elite athletes, indicating highly synchronized mechanical behavior between extensors and flexors, and (2) markedly higher variability-based velocity coordination (RMTD_cV) in the dominant leg, suggesting enhanced modulation of contraction dynamics during high-speed actions.

## 4. Discussion

The primary aim of this study was to determine whether sport expertise differentiates twitch-derived individual TMG parameters and contractile ratio indexes between knee extensors and flexors. Unlike classical EMG-based coactivation, which reflects simultaneous neural activation, the present indexes represent ratios between involuntary contractile parameters and therefore describe mechanical coordination between antagonistic muscle groups. The results suggest that elite performance in karate is characterized by limb-specific mechanical coordination strategies: temporally efficient contractile interplay in the supportive leg and velocity-modulated mechanical coupling in the executive leg.

### 4.1. Individual TMG Parameters

Differences in Tc and TcT variables were observed between all karate subgroups and the non-athlete control group (CG), as well as between EK and the NK and BK subgroups, suggesting training-related adaptations in the contractile properties of knee extensor agonist muscles [23]. Variations in these parameters between dominant and non-dominant limbs indicate that karate athletes generally exhibit greater explosive characteristics than CG, potentially reflecting differences in muscle fiber composition or neuromuscular activation patterns [27]. In support of this interpretation, international-level karate athletes have been shown to recruit a greater number of fast motor units in the quadriceps during the attack phase of Mae geri kick compared with national-level athletes [28]. Differences observed between BK and CG in the VL of both limbs for Tc and TcT (Table 2 and Table 3) may reflect cumulative effects of repeated training exposure [29], consistent with results from the study by Jemili et al. [9] reporting reductions in quadriceps contraction time following intensive karate training. Collectively, these findings are compatible with the notion that structured, sport-specific training is associated with bilateral neuromechanical adaptations characterized by faster muscle contraction dynamics [30,31]. During karate striking actions, the rectus femoris is specifically involved in both functions by reducing the time the foot is lifted from the ground and increasing the velocity of tibial extension [32,33]. Given that karate strikes require the coordinated execution of hip flexion (jap. hiki ashi) and knee extension, the RF displays contraction characteristics that differ from those of the monoarticular knee extensors (VL and VM). Accordingly, this muscle demonstrates both reactive and contraction velocity properties, reflected in statistically significant differences in TcT in the non-dominant leg (Table 3) and Tc in the dominant leg (Table 2), as well as significant RMTD differences between EK and BK and between EK and CG in both dominant and non-dominant limbs (Table 2 and Table 3).

Higher RMTD values of the knee flexors BF and ST in the dominant leg observed in EK and NK compared with CG (Table 4) indicate pronounced contraction velocity characteristics of the hamstring, likely reflecting sport-specific learning strategies developed to regulate leg strikes in accordance with karate competition rules [28,34]. On the other hand, the only statistically significant difference in the RMTD variable of the non-dominant leg was observed in the BF muscle between the EK and CG (Table 5). These RMTD differences in antagonist muscles appear to be based on long-term training experience and represent a centrally programmed anticipatory control mechanism that modulates strike force and contributes to knee joint stabilization prior to impact [9]. Consistent with this interpretation, kumite athletes exhibited a high capacity to selectively activate or inhibit antagonistic muscles to stabilize and protect the joint during both lower and upper limb striking actions [35]. Similar patterns of muscle behavior have been found in research by Sbriccoli et al. [11]. Elite karateka demonstrate greater antagonist activation than lower-level competitors during Mae geri, reflecting an adopted strategy to enhance control of the striking leg. Furthermore, Pozo et al. [28] suggested that elevated eccentric activation at the end of the attack phase may function as a pre-activation strategy that anticipates knee flexion and hip extension during the recovery phase.

**Table 4:** Descriptive statistics of measured variables (Tc, TcT, and RMTD) for the knee flexor muscles of the dominant leg according to the examined subgroups.

	GROUPS				ANOVA			
	Karate Elite	Karate National	Karate Basic	Control Group	F	Sig.	Part. Eta^2^	Power
BF_Tc_D	24.51 ± 3.49	35.17 ± 10.24	33.60 ± 10.41	34.46 ± 9.33	2.388	0.081	0.132	0.561
BF_TcT_D	46.66 ± 4.18	58.13 ± 11.95	56.67 ± 12.96	61.37 ± 15.85	2.245	0.095	0.125	0.533
BF_RMTD_D	0.227 ± 0.064 *	0.187 ± 0.038	0.173 ± 0.080	0.145 ± 0.049	3.425	0.025	0.179	0.735
ST_Tc_D	38.19 ± 15.23	42.89 ± 9.88	37.81 ± 10.97	34.85 ± 13.48	0.892	0.452	0.054	0.230
ST_TcT_D	61.04 ± 17.58	67.45 ± 11.30	61.17 ± 12.70	60.24 ± 15.34	0.739	0.534	0.045	0.196
ST_RMTD_D	0.202 ± 0.052 *	0.218 ± 0.046 ^§§^	0.185 ± 0.078 ^Ø^	0.124 ± 0.054	6.504	0.001	0.293	0.958

Note: Statistical significance between the groups was marked as follows: EK and CG—*; NK and CG—§; BK and CG—Ø. The number of symbols indicates the strength of statistical significance. Example: *p* < 0.050 *; *p* < 0.010 **. Parameters are expressed in ms.

**Table 5:** Descriptive statistics of measured variables (Tc, TcT, and RMTD) for the knee flexor muscles of the non-dominant leg according to the examined subgroups.

	GROUPS				ANOVA			
	Karate Elite	Karate National	Karate Basic	Control Group	F	Sig.	Part. Eta^2^	Power
BF_Tc_ND	26.77 ± 8.00 ^¥^	42.81 ± 13.03	32.31 ± 11.82	36.31 ± 10.04	3.854	0.015	0.197	0.789
BF_TcT_ND	47.97 ± 8.00 ^¥^	66.21 ± 14.71	54.87 ± 13.25	59.37 ± 14.67	3.392	0.025	0.178	0.730
BF_RMTD_ND	0.219 ± 0.061 *	0.168 ± 0.054	0.182 ± 0.081	0.135 ± 0.058	3.083	0.036	0.164	0.684
ST_Tc_ND	28.97 ± 9.74 ^@^ **	41.49 ± 13.05	43.79 ± 10.51	47.25 ± 7.19	5.070	0.004	0.245	0.896
ST_TcT_ND	50.85 ± 10.93 ^@^ **	65.07 ± 15.37	67.51 ± 10.95	72.52 ± 7.78	5.533	0.002	0.261	0.922
ST_RMTD_ND	0.229 ± 0.056	0.204 ± 0.051	0.183 ± 0.083	0.145 ± 0.083	2.863	0.047	0.155	0.648

Note: Statistical significance between the groups was marked as follows: EK and NK—¥; EK and BK—@; EK and CG—*. The number of symbols indicates the strength of statistical significance. Example: *p* < 0.050 *; *p* < 0.010 **. Parameters are expressed in ms.

### 4.2. Contractile Ratio Indexes

Elite karate athletes exhibited markedly lower temporal contractile ratio indexes (17.13% for Tc and 9.72% for TcT) in the non-dominant leg compared with other groups (Table 6 and Table 7). These findings indicate a high degree of temporal similarity between extensors and flexors in the supportive limb. Significant differences are found between EK and NK (*p* = 0.003) for the CRI_Tc_AVG_ND (Table 6) and between EK and NK (*p* = 0.002) for the CRI_TcT_AVG_ND (Table 7). Functionally, this pattern may reflect optimized mechanical modulation supporting dynamic stability during single-leg stance rather than increased joint stiffness. For example, a recent study conducted three-dimensional analysis of the support leg during a roundhouse kick and showed distinct joint and loading mechanics in the support leg according to athlete weight category, underscoring the support leg’s critical biomechanical role [36]. Balance performance has been shown to be superior in elite karate athletes: higher-ranked fighters exhibit superior postural stability in the supporting (non-dominant) leg, enabling effective horizontal displacement toward the opponent while the dominant leg is elevated during offensive actions [12]. Consistently, previous research comparing elite and amateur karate practitioners [37] reported that elite athletes display more advanced control of dynamic balance, particularly during kicking techniques performed with one leg raised. This enhanced balance control has been attributed to prolonged exposure to complex motor demands throughout high-level training, including repeated shifts of the center of mass, rotational movements, and single-leg stances. Such tasks require a high degree of neuromuscular coordination of the non-dominant supporting leg [37]. The sub-elite group (NK) had relatively better individual parameters in the agonist extensor muscles of the non-dominant leg (Table 3), whereas elite competitors (EK) demonstrated superior mechanical coordination between agonist and antagonist muscle groups—CRI_Tc and CRI_TcT (Table 6 and Table 7). This implies that elite status is characterized not merely by faster contraction times or greater force but by optimal temporal mechanical coordination between opposing muscle groups.

In contrast, elite athletes showed the highest velocity-related contraction ratio index (CRI_RMTD_Cv_H/Q_D) in the dominant leg (63.66%), significantly above other groups. More precisely, differences are found between EK and NK (*p* = 0.003), EK and BK (*p* = 0.002), and EK and CG (*p* = 0.009)—Table 8. This suggests a distinct mechanical profile characterized by enhanced modulation of contraction velocity between antagonistic muscle groups. During high-speed kicking actions requiring rapid acceleration followed by controlled deceleration, such velocity-sensitive mechanical interplay may represent a task-specific adaptation. During a karate kick, the dominant leg must not only produce high angular acceleration but also decelerate rapidly [35] to maintain technique control in accordance with competition regulations, which strictly penalize excessive contact or uncontrolled execution [34]. This dual requirement for speed and precision suggests that elevated variability in RMTD may reflect a neuromechanical adaptation that facilitates both explosive initiation and fine-tuned braking of the movement [38]. A higher level of the coactivation index between the knee extensors and flexors of the dominant (executive) leg has been reported when comparing senior and junior national-level karate athletes [13]. In that study, electromyography was used to examine the activity of the vastus lateralis (VL) and biceps femoris (BF) during the execution of the mawashi-geri kick. In addition to displaying a higher coactivation index, the senior athletes demonstrated a greater BF activation index in its antagonistic role during the knee-extension phase compared with the junior group. This enhanced antagonist activation was interpreted as evidence of “peripheral and central nervous system remodeling directly dependent on the level of skill” [13].

In addition to coordination-oriented approaches, lower-limb muscle imbalances are commonly assessed using a range of complementary methodologies that provide insight into performance-related neuromuscular function. Inter-limb asymmetries are frequently quantified through unilateral performance tests such as countermovement jump (CMJ) [39], single-leg hop tests [40], or isokinetic dynamometry [41], where asymmetry indexes are calculated based on force, power, or torque outputs. Agonist–antagonist imbalances are typically evaluated using isokinetic strength ratios (e.g., hamstring-to-quadriceps ratio), which have been associated with both performance capacity and injury risk. Electromyography (EMG) further enables the assessment of activation timing and magnitude, offering insight into neural coordination patterns during dynamic tasks. More recently, tensiomyography has been applied to detect contractile property differences between limbs and between muscle groups, using parameters such as contraction time (Tc), delay time (Td), and maximal displacement (Dm) to identify functional asymmetries [42]. Studies have shown that these TMG-derived asymmetries may relate to differences in explosive performance, such as jump height and rate of force development [43], particularly when combined with dynamic assessments. Therefore, the twitch-derived coordination indexes proposed in the present study may be interpreted as part of a broader framework of neuromuscular imbalance assessment, providing complementary information on intrinsic contractile behavior across different sport populations, as shown in the research by Cesanelli et al. [44].

EMG-based approaches quantify neural coactivation by assessing simultaneous activation of agonist and antagonist muscles during voluntary movement [11,13], providing high ecological validity but being influenced by task execution and central drive. In contrast, TMG assesses isolated muscle contractile properties under involuntary conditions, offering high standardization and muscle-specific insight, but limited direct transfer to dynamic performance. Therefore, these methods should be viewed as complementary rather than interchangeable.

### 4.3. Practical Applications

The present findings provide several implications for karate training and performance optimization. First, the lower temporal contractile ratio indexes observed in the non-dominant leg of elite athletes highlight the importance of developing balanced and synchronized contractile behavior between knee extensors and flexors in the supporting limb. Coaches may therefore incorporate stability-oriented and unilateral training drills aimed at improving neuromuscular control during single-leg stance conditions. Second, the elevated variability in velocity-related coordination in the dominant leg suggests that elite athletes possess a refined ability to modulate contraction speed during explosive actions. Training programs should therefore include exercises that combine rapid force production with controlled deceleration (e.g., ballistic kicks with braking emphasis and eccentric hamstring work). Finally, twitch-derived contractile ratio indexes may serve as a complementary diagnostic tool for monitoring neuromuscular adaptations, identifying asymmetries, and individualizing training interventions in combat sports.

### 4.4. Study Limitations

As a cross-sectional study, these findings represent a snapshot of neuromuscular coordination at a single time point. Future longitudinal or interventional research is needed to clarify how these coordination patterns evolve with training and whether targeted neuromuscular programs can modify contraction ratio indexes to enhance performance. Importantly, these findings should not be interpreted as evidence of greater neural coactivation. Because the measurements were obtained under involuntary twitch conditions at a fixed joint angle (120° knee flexion), the indexes reflect intrinsic contractile behavior and intermuscular mechanical relationships rather than central motor drive. Since muscle force is determined by its length and velocity [45], and during dynamic tasks, the length and velocity of the muscles vary differently, these two factors might limit the use of CRI based exclusively on the TMG signals. Importantly, twitch-derived coordination indexes should not be interpreted as direct measures of neural coactivation, but rather as indirect indicators of the mechanical interplay between muscles. Moreover, integrating TMG with surface EMG, kinetic/kinematic analyses, and muscle synergy modeling would further strengthen mechanistic interpretations. The absence of sport-specific movement tasks limits the ecological validity of the findings, and future studies should integrate TMG with dynamic assessments (e.g., EMG or force plate analysis). Additionally, expanding research to include female athletes and various karate styles would improve generalizability. Finally, the relatively small sample size of the elite group (*n* = 7) should be acknowledged as a limitation. Although this reflects the practical difficulty of recruiting high-level athletes, it may limit the generalizability of the findings. Future studies with larger samples and multi-center collaboration are recommended to confirm these results.

## 5. Conclusions

This study demonstrates that sport expertise in karate is associated with distinct twitch-derived mechanical coordination patterns between knee extensors and flexors. Elite athletes exhibit lower temporal contractile ratio indexes in the non-dominant (supportive) leg and higher velocity-related contractile ratio indexes in the dominant (executive) leg. Markedly lower contraction ratio indexes for contraction time and total contraction time in the non-dominant leg probably indicate a refined temporal interplay between quadriceps and hamstring muscles that supports dynamic stability during complex single-leg actions. On the other side, the highest contractile ratio for the coefficient of variation of contraction velocity in the dominant leg likely reflects a neuromechanical adaptation that facilitates explosive yet precisely controlled kicking actions—movements that must accelerate rapidly and then decelerate efficiently to meet the technical and regulatory demands of point karate. The results extend the current understanding of karate practitioners’ neuromechanical adaptation by introducing twitch-derived contractile ratios as potential indicators of sport-specific mechanical coordination.

## Figures and Tables

**Table 1:** Descriptive characteristics of the study participants.

Variables	Karate Elite		Karate National		Karate Basic		Control Group	
	Mean	SD	Mean	SD	Mean	SD	Mean	SD
Age	28.67	2.66	21.79	3.12	20.13	0.96	26.62	3.28
BH (cm)	184.63	2.76	180.56	9.07	183.44	8.44	179.50	5.96
BM (kg)	86.43	7.02	77.12	6.89	80.88	9.17	82.58	13.82
BMI (kg/m^2^)	25.38	2.38	23.67	1.61	24.05	2.27	25.52	3.46

Note: BH—body height; BM—body mass; BMI—body mass index; SD—standard deviation.

**Table 2:** Descriptive statistics of measured variables (Tc, TcT, and RMTD) for the knee extensor muscles of the dominant leg according to the examined subgroups.

	GROUPS				ANOVA			
	Karate Elite	Karate National	Karate Basic	Control Group	F	Sig.	Part. Eta^2^	Power
RF_Tc_D	25.15 ± 2.19 *	29.04 ± 5.19	28.11 ± 5.95	32.07 ± 6.56	2.794	0.049	0.151	0.636
RF_TcT_D	47.52 ± 2.53	52.77 ± 7.03	49.99 ± 6.52	54.25 ± 8.22	2.129	0.109	0.120	0.509
RF_RMTD_D	0.287 ± 0.075 ^@^ **	0.213 ± 0.064	0.184 ± 0.081	0.171 ± 0.007	4.378	0.008	0.218	0.843
VL_Tc_D	22.91 ± 2.67	24.09 ± 3.06	21.99 ± 4.25 ^Ø^	26.01 ± 3.84	2.796	0.049	0.151	0.637
VL_TcT_D	44.60 ± 3.59	45.41 ± 2.76	43.73 ± 7.10 ^Ø^	50.27 ± 5.77	3.736	0.017	0.193	0.775
VL_RMTD_D	0.246 ± 0.066	0.208 ± 0.061	0.228 ± 0.121	0.197 ± 0.107	0.451	0.718	0.028	0.134
VM_Tc_D	27.37 ± 2.90	22.89 ± 1.68	24.82 ± 3.89	27.26 ± 9.03	2.467	0.074	0.136	0.577
VM_TcT_D	52.99 ± 8.55 ^¥^	44.03 ± 2.06 ^§^	47.38 ± 5.22	50.36 ± 9.92	3.830	0.016	0.196	0.786
VM_RMTD_D	0.289 ± 0.069	0.342 ± 0.089	0.295 ± 0.119	0.259 ± 0.087	1.957	0.133	0.111	0.473

Note: Statistical significance between the groups was marked as follows: EK and NK—¥; EK and BK—@; EK and CG—*; NK and CG—§; BK and CG—Ø. The number of symbols indicates the strength of statistical significance. Example: *p* < 0.050 *; *p* < 0.010 **. Parameters are expressed in ms.

**Table 3:** Descriptive statistics of measured variables (Tc, TcT, and RMTD) for the knee extensor muscles of the non-dominant leg according to the examined subgroups.

	GROUPS				ANOVA			
	Karate Elite	Karate National	Karate Basic	Control Group	F	Sig.	Part. Eta^2^	Power
RF_Tc_ND	23.49 ± 3.16	26.66 ± 6.13	28.40 ± 8.40	31.82 ± 5.66	2.529	0.069	0.139	0.588
RF_TcT_ND	45.24 ± 4.38 *	48.21 ± 8.08	50.82 ± 10.23	56.70 ± 7.62	3.266	0.029	0.172	0.712
RF_RMTD_ND	0.304 ± 0.076 ^@@^ *	0.218 ± 0.078	0.176 ± 0.086	0.176 ± 0.070	4.597	0.007	0.227	0.862
VL_Tc_ND	22.23 ± 1.69	23.93 ± 3.92	21.57 ± 2.60 ^ØØ^	25.44 ± 3.28	4.489	0.008	0.223	0.853
VL_TcT_ND	43.04 ± 2.49	45.06 ± 5.73	42.76 ± 4.22 ^ØØ^	48.60 ± 4.64	4.547	0.007	0.225	0.858
VL_RMTD_ND	0.270 ± 0.055	0.209 ± 0.060	0.205 ± 0.092	0.209 ± 0.078	1.423	0.248	0.083	0.353
VM_Tc_ND	25.73 ± 2.27	22.85 ± 2.47 ^§^	24.73 ± 7.68	27.88 ± 5.79	2.700	0.056	0.147	0.620
VM_TcT_ND	46.92 ± 3.01	43.76 ± 3.13 ^§^	45.64 ± 8.72	50.80 ± 7.19	3.739	0.017	0.193	0.776
VM_RMTD_ND	0.310 ± 0.064	0.336 ± 0.123 ^§^	0.260 ± 0.078	0.221 ± 0.095	4.164	0.011	0.210	0.823

Note: Statistical significance between the groups was marked as follows: EK and BK—@; EK and CG—*; NK and CG—§; BK and CG—Ø. The number of symbols indicates the strength of statistical significance. Example: *p* < 0.050 *; *p* < 0.010 **. Parameters are expressed in ms.

**Table 6:** Results of the Contractile Ratio Indexes (CRI) of the dominant and non-dominant extensor and flexor muscles in the knee joint for the parameter contraction time (Tc). The results were obtained using Formulas (1) and (2).

	GROUPS				ANOVA			
	Karate Elite	Karate National	Karate Basic	Control Group	F	Sig.	Part. Eta^2^	Power
CRI_Tc_AVG_D	25.79	53.76	44.64	25.75	2.118	0.111	0.119	0.507
CRI_Tc_cV_D	261.29	153.77	69.40	636.17	0.585	0.628	0.036	0.162
CRI_Tc_AVG_ND	17.13 ^¥¥^	73.38	56.13	47.12	4.949	0.005	0.240	0.888
CRI_Tc_cV_ND	252.16	120.08	125.64	71.39	0.682	0.568	0.042	0.183

Note: Statistical significance between the groups was marked as follows: EK and NK—¥. The number of symbols indicates the strength of statistical significance. Example: *p* < 0.050 *; *p* < 0.010 **.

**Table 7:** Results of the Contractile Ratio Indexes (CRI) of the dominant and non-dominant extensor and flexor muscles in the knee joint for the parameter total contraction time (TcT). The results were obtained using Formulas (3) and (4).

	GROUPS				ANOVA			
	Karate Elite	Karate National	Karate Basic	Control Group	F	Sig.	Part. Eta^2^	Power
CRI_TcT_AVG_D	12.43	32.22	25.69	18.95	1.699	0.180	0.098	0.416
CRI_TcT_Cv_D	300.03	198.14	65.49	99.04	1.204	0.319	0.071	0.302
CRI_TcT_AVG_ND	9.72 ^¥¥^	44.08	33.05	26.58	5.229	0.003	0.250	0.906
CRI_TcT_Cv_ND	227.42	117.54	202.96	73.71	0.679	0.569	0.042	0.182

Note: Statistical significance between the groups was marked as follows: EK and NK—¥. The number of symbols indicates the strength of statistical significance. Example: *p* < 0.050 *; *p* < 0.010 **.

**Table 8:** Results of the Contractile Ratio Indexes (CRI) of the dominant and non-dominant extensor and flexor muscles in the knee joint for the parameter rate of muscle tension development (RMTD). The results were obtained using Formulas (5) and (6).

	GROUPS				ANOVA			
	Karate Elite	Karate National	Karate Basic	Control Group	F	Sig.	Part. Eta^2^	Power
CRI_RMTD_AVG_D	−20.69	−18.74	−23.37	−34.03	1.535	0.218	0.089	0.378
CRI_RMTD_Cv_D	63.66 ^¥¥ @@^ **	−45.42	−46.87	−35.16	5.835	0.002	0.271	0.936
CRI_RMTD_AVG_ND	−21.20	−4.68	0.34	−27.34	2.466	0.074	0.136	0.576
CRI_RMTD_Cv_ND	17.69	213.56	36.42	1601.42	0.843	0.477	0.051	0.219

Note: Statistical significance between the groups was marked as follows: EK and NK—¥; EK and BK—@; EK and CG—*. The number of symbols indicates the strength of statistical significance. Example: *p* < 0.050 *; *p* < 0.010 **. Parameters are expressed in ms.

## Data Availability

The raw data supporting the conclusions of this article will be made available by the authors on request.
